# HLA specificities are associated with prognosis in IGHV-mutated CLL-like high-count monoclonal B cell lymphocytosis

**DOI:** 10.1371/journal.pone.0172978

**Published:** 2017-03-01

**Authors:** María García-Álvarez, Miguel Alcoceba, Miriam López-Parra, Noemí Puig, Alicia Antón, Ana Balanzategui, Isabel Prieto-Conde, Cristina Jiménez, María E. Sarasquete, M. Carmen Chillón, María Laura Gutiérrez, Rocío Corral, José María Alonso, José Antonio Queizán, Julia Vidán, Emilia Pardal, María Jesús Peñarrubia, José M. Bastida, Ramón García-Sanz, Luis Marín, Marcos González

**Affiliations:** 1 Department of Hematology, University Hospital of Salamanca (HUS-IBSAL), Salamanca, Spain; 2 Cooperative Working Group on Lymphomas and Lymphoproliferative Disorders of the Castilla y León Society of Hematology and Hemotherapy (SCLHH), Castilla y León, Spain; 3 CIBERONC, Madrid, Spain; 4 Cytometry Service-NUCLEUS, Department of Medicine, Cancer Research Center (IBMCC-CSIC/USAL) and IBSAL (University of Salamanca), Salamanca, Spain; Universita degli Studi di Napoli Federico II, ITALY

## Abstract

**Introduction:**

Molecular alterations leading progression of asymptomatic CLL-like high-count monoclonal B lymphocytosis (hiMBL) to chronic lymphocytic leukemia (CLL) remain poorly understood. Recently, genome-wide association studies have found 6p21.3, where the human leukocyte antigen (HLA) system is coded, to be a susceptibility risk region for CLL. Previous studies have produced discrepant results regarding the association between HLA and CLL development and outcome, but no studies have been performed on hiMBL.

**Aims:**

We evaluated the role of HLA class I (-A, -B and -C) and class II (-DRB1 and -DQB1) in hiMBL/CLL susceptibility, hiMBL progression to CLL, and treatment requirement in a large series of 263 patients diagnosed in our center with hiMBL (n = 156) or Binet A CLL (n = 107).

**Results:**

No consistent association between HLA specificities and hiMBL or CLL susceptibility was found. With a median follow-up of 7.7 years, 48/156 hiMBLs (33%) evolved to asymptomatic CLLs, while 16 hiMBLs (10%) and 44 CLLs (41%) required treatment. No HLA specificities were found to be significantly associated with hiMBL progression or treatment in the whole cohort. However, within antigen-experienced immunoglobulin heavy-chain (IGHV)-mutated hiMBLs, which represents the highest proportion of hiMBL cases (81%), the presence of HLA-DQB1*03 showed a trend to a higher risk of progression to CLL (60% vs. 26%, P = 0.062). Moreover, HLA-DQB1*02 specificity was associated with a lesser requirement for 15-year treatment (10% vs. 36%, P = 0.012).

**Conclusion:**

In conclusion, our results suggest a role for HLA in IGHV-mutated hiMBL prognosis, and are consistent with the growing evidence of the influence of 6p21 on predisposition to CLL. Larger non-biased series are required to enable definitive conclusions to be drawn.

## Introduction

Chronic lymphocytic leukemia (CLL)-like monoclonal B-cell lymphocytosis (MBL) is an asymptomatic monoclonal expansion defined according to the WHO 2008 classification [1] and the International Working Group on CLL (IWCLL) guidelines [2, 3] as the presence of CLL-phenotype B cells at a concentration of <5x10^9^/L and without disease-related symptoms, such as cytopenias, lymphadenopathies or organomegaly.

Two groups of CLL-like MBL patients can be differentiated. A small fraction of MBL cases (~10%) are described as high-count MBL (hiMBL), being diagnosed during the characterization of otherwise asymptomatic lymphocytosis with a whole lymphocyte count over 3.5x10^9^/L [4, 5]. It is assumed to be a precursor state of CLL, with a progression rate to CLL that requires treatment of ~1–2% per year [3–7]. All other MBL cases are accidentally found by screening individuals with a normal blood cell count, and are defined as “low-count MBL” (loMBL), with very low risk of progression to CLL [8, 9].

Previous studies indicate that most of the usual clinical variables (including age, hemoglobin levels) are not correlated with risk of disease progression or requirement for treatment in hiMBL [4–7, 10–12]. Recently, the CLL phenotype lymphocyte count in peripheral blood has been related to higher progression to CLL/SLL [4], while the absolute B-cell count, unmutated immunoglobulin heavy-chain variable region (IGHV) status, presence of trisomy 12 or del17p13, and CD38 expression ≥30% are known to be independent prognostic factors of low 10-year treatment-free survival (TFS) [4, 7, 11–13].

In recent years, genome-wide association studies (GWAS) have identified the 6p21.3 region as a susceptibility risk region for familial and sporadic CLLs [14–17]. The human leukocyte antigen (HLA) system, situated in this region, plays a role in antitumor immune responses and lymphoma-cell apoptosis [18], and could therefore be essential for the control of neoplasias. In this context, previous studies have established that there is a relationship between HLA polymorphisms and susceptibility to hematological disorders [19–23]. Focusing on CLL, previous reports have related various HLA specificities to susceptibility to CLL [20, 24] and worse prognosis [25, 26]. Despite the evidence for the influence of this region on CLL evolution and behavior, there is little information available concerning its role in hiMBL.

In the present retrospective study, we have evaluated whether the HLA class I (-A, -B and -C) and class II (-DRB1 and -DQB1) polymorphisms are associated with (i) the incidence of hiMBL and CLL; (ii) progressive lymphocytosis; (iii) progression to CLL; (iv) treatment-free survival (TFS); and (v) overall survival (OS), in a series of 156 hiMBL individuals.

## Patients and methods

### Patient characteristics

We included a non-selected (unbiased) series of patients analyzed between 1992 and 2014 with the following inclusion criteria: 1) B-lymphocyte expansions with CLL phenotype confirmed in peripheral blood by flow cytometry (FCM) according to the 2008 WHO classification [1] and the IWCLL [3]; 2) clinical and follow-up data, as well as DNA sample were available. Exclusion criteria were: 1) Binet B or C clinical stages, 2) loMBL; 3) familial cases, and 4) patients HLA-typed as possible candidates for allogeneic stem-cell transplantation out of the former criteria.

Our study included 263 cases harboring B-lymphocyte expansions with CLL phenotype. Within this series, 156 individuals with a whole lymphocyte count greater than 3.5x10^9^/L (local normal range, 1.2–3.5x10^9^/L) were diagnosed with hiMBL (<5x10^9^/L clonal B cell count, measured by FCM) during the characterization of an otherwise asymptomatic lymphocytosis and were compared with 107 CLL cases of Binet stage A.

The control population for HLA-A, HLA-B and HLA-DRB1 loci consisted of 1940 healthy donor individuals from the Spanish Castilla y León registry for stem cell transplant donors matched with the cases by gender, age, and ethnicity [27]. In addition, HLA-C and HLA-DQB1 were typed in 200 of these donors [23].

This study was approved by the local Ethics Review Committee in accordance with Spanish law. Written informed consent was obtained from all participants.

### DNA extraction and HLA typing

DNA from peripheral blood and bone marrow samples was isolated using DNAzol reagent (MRC, Cincinnati, OH) or the Maxwell® 16 System (Promega, Madison, WI, USA) [28]. Low-resolution (two digits) typing of HLA class I (A, B and C) and HLA class II (DRB1 and DQB1) was carried out using the polymerase chain reaction (PCR) reverse sequence-specific oligonucleotide and Luminex XYP technology (Tepnel Lifecodes Corporation, Stamford, CT), according to the standards of the European Federation of Immunogenetics (http://www.efiweb.eu). PCR sequence-specific primer methods (Dynal Biotech, Oslo, Norway) were also used as necessary for HLA-A, -B, -C, -DRB1, and -DQB1 loci. Homozygosis was confirmed by familial studies or by using two alternative methods.

### Analysis of IGHV mutation status

IGH rearrangements were amplified according to the BIOMED-2 Concerted Action protocols, in the standardization of which our group has participated [29, 30] The subsequent sequencing analysis of the monoclonal immunoglobulin rearrangement has been described before [30, 31]. According to the European Research Initiative on CLL (ERIC) recommendations, the sequences obtained were aligned to ImMunoGeneTics database (IMGT, http://www.imgt.org/) and analyzed using IMGT/V-Quest software. Sequences with a germline homology of at least 98% were considered to be unmutated, while those with less than 98% homology were considered to be sequences with somatic hypermutation (SHM) or to be mutated [31, 32].

### Interphase FISH analysis

Interphase FISH analysis was performed on peripheral blood samples obtained at diagnosis to detect del(11)(q22.3) (*ATM*), del(13)(q14), del(17)(p13) (*TP53*), and trisomy 12. CD19+ selection was performed in most hiMBL samples (68%), and in all hiMBL and CLL cases with low tumour percentage to rule out false negatives. These were evaluated using commercially available probes according to the manufacturer’s protocols, as previously described (Vysis/Abbott Co., Downers Grove, IL, USA) [33]. The threshold used to designate FISH alterations was 10%.

### Definitions and statistical analyses

HLA allele frequencies were estimated by an expectation-maximization algorithm using the Arlequin software package (version 3.5.1.2) [34]. The Hardy-Weinberg equilibrium was tested by applying a modified hidden Markov chain with a 100,000 step-length approach and 10,000 dememorization steps, as implemented in the Arlequin program. Allele frequencies between populations were compared with the two-sided Fisher’s exact test in GraphPad Prism 4.0 (GraphPad Software, San Diego, CA). The strength of associations was estimated as odds ratios (ORs) with 95% confidence intervals (CIs), calculated by the Cornfield method (values of *P*<0.05 were considered statistically significant). *P*-values were Bonferroni-corrected (*Pc*) to take into account HLA-locus multiple testing.

We explored the following clinical endpoints: (i) progression from hiMBL to asymptomatic CLL, defined as the presence of a stable >5x10^9^/L clonal B-cell count; (ii) progressive lymphocytosis, defined as lymphocyte count that was more than twice the count at presentation and remained at this level or increased at subsequent assessments[6]; (iii) treatment-free survival (TFS) according to IWCLL guidelines, defined as the time from date of diagnosis to date of first treatment [3]; and (iv) OS, defined as the time from date of diagnosis of hiMBL to death from any cause [3]. End-points were assessed on the date of the last patient contact; the most recent follow-up was in December 2015. In all cases, surviving patients were censored at last follow-up.

The following variables were included in the survival analysis in addition to the presence of the various HLA polymorphisms: sex, age, absolute lymphocyte count, CLL-lymphocyte count, IGHV status, β2-microglobulin level, and FISH analyses. Due to the low prevalence of del11q, del17p, and +12 at diagnosis in hiMBL and CLL patients (Table 1), these intermediate/high-risk genetic lesions were grouped and compared with del13q/normal cytogenetics. FISH variables were excluded from multivariate analyses due to there being insufficient patients with available data.

**Table 1:** Biological and clinical characteristics of hiMBL (n = 156) and Binet A CLL (n = 107) cohorts at diagnosis.

Variable	hiMBL n (%)	CLL n (%)	*P*
Median follow-up, months (range)	73 (1–307)	95 (3–250)	0.069
**Clinical variables at diagnosis**			
Age [years, median (range)]	68 (30–89)	63 (29–86)	0.0002
≤60	25 (16)	47 (44)	6.9x10^-7^
Sex			
Male	86 (55)	66 (62)	0.18
WBC (mean ± SD; x10^9^/L)	11.8±2.5	22.2±13.1	1.5x10^-12^
Absolute lymphocyte count (mean ± SD; x10^9^/L)	7.0±1.7	16.6±12.3	4.2x10^-12^
CLL-type cells (mean ± SD; x10^9^/L)	3.1±1.2	12.0±11.1	1.4x10^-12^
β2-microglobulin >3.5 mg/L*	17 (11)	12 (14)	0.33
Treated	16 (10)	44 (41)	6.7x10^-9^
**Biological variables at diagnosis**			
IGHV homology [mean (25th-75th percentiles)]	94.0 (91.6–96)	96 (93–100)	2.7x10^-5^
IGHV unmutated (homology ≥98%)*	28 (19)	46 (44)	1.5x10^-5^
FISH*			
Normal	53 (40)	41 (43)	0.39
del(11)(q22.3)	2 (2)	5 (5)	0.12
+12	19 (15)	9 (9)	0.18
del(13)(q14)	61 (47)	43 (45)	0.51
del(17)(p13)	0 (-)	4 (4)	0.031

* β2-microglobulin was available in 154 (99%) hiMBL and 87 (81%) Binet A CLL cases; IGHV status was available in 149 (96%) hiMBL and 105 (98%) Binet A CLL cases. FISH analyses were performed in 131 (84%) hiMBL and 95 (89%) Binet A CLL cases.

The associations between variables were analyzed by the χ^2^-square test for categorical variables and by Student’s t-test or logistic regression for continuous variables. Cut-off values for relevant continuous variables were estimated using ROC curves. Survival was estimated according to the Kaplan–Meier method, and the two-sided log-rank test was used to test univariate associations between variables. Subsequently, all variables for which there was some indication of a significant association with progressive hiMBL and TFS in the univariate test (*P*<0.1) were included in the multivariate analysis using a Cox regression model. Differences were considered to be statistically significant for values of *P*<0.05. These analyses were performed using SPSS (SPSS 20.0, IBM Corp, Armonk, NY, USA).

## Results

### Clinical and biological variables associated with hiMBL and CLL

The biological and clinical characteristics of hiMBL and Binet A CLL are compared in Table 1. Unmutated IGHV (IGHV homology ≥98%) status (19% vs. 43%, *P*<0.001) and young age (≤60 years, 16% vs. 44%, *P*<0.001) were significantly lower in hiMBL patients than Binet A CLL. The way in which both entities are diagnosed may explain why we found a younger age for CLL than for hiMBL patients, since the diagnosis of a CLL is easy diagnosed by screening hemogram while MBL is only found after directed flow cytometry analysis. No differences were found with respect to gender or cytogenetics between the cohorts. With a median follow-up of 7.7 years, 56 hiMBL (38%) cases presented progressive lymphocytosis, 48 hiMBL cases (33%) progressed to CLL, while 16 hiMBL (10%) and 44 CLL (41%) patients received treatment. No patients were lost to follow-up. Due to the strong correlation between progressive lymphocytosis and hiMBL progression to CLL (138/146 of the cases (95%), p<0.001), we decided to exclude the progressive lymphocytosis in the HLA association analyses.

When ROC curves for progression rates from hiMBL to CLL were calculated a cut-off of >2.5x10^9^/L clonal B-cell count at diagnosis was established. In the multivariate analysis, patients with >2.5x10^9^/L clonal B-cell counts (74% vs. 17%, *P*<0.001, HR: 3.9, 95% CI: 1.5–10.0), and unmutated IGHV status (100% vs. 54%, *P*<0.001, HR: 2.3, 95% CI: 1.2–4.3) had greater 15-year progression to CLL (S1 Table).

### Association of HLA with progression to CLL

HLA polymorphism frequencies of hiMBL patients were consistent in all cases with the Hardy-Weinberg equilibrium. The HLA-A, -B and -DRB1 allele frequencies of the control population from our geographic region have been described elsewhere [27]. The HLA-C and HLA-DQB1 polymorphism frequencies were similar to those of other Iberian populations [23].

To identify those HLA polymorphisms related to progression from hiMBL to CLL, we first made a stringent comparison of HLA frequencies in those hiMBLs without progression to CLL with a minimal follow-up of three years (non-pMBL, n = 78) with those of the combined group of Binet A CLL and hiMBL with progression to CLL (pMBL/CLL, n = 144). The HLA alleles found to be significantly different between the two groups were then analyzed in the whole cohort of hiMBL (n = 156) to identify their possible association with progression to CLL. All allelic frequencies and the rate of HLA homozygosity were systematically examined and compared among non-pMBL, pMBL/CLL, and healthy control groups (S2 Table).

Allele frequencies differed significantly between pMBL/CLL and healthy control individuals, whereby we found a higher frequency of HLA-A*11 (14% vs. 7%, *P*<0.001, *Pc* = 0.008), HLA-DRB1*03 (18% vs. 12%, *P* = 0.019, *Pc* = 0.180), and HLA-DQB1*02 (33% vs. 27%, *P* = 0.029, *Pc* = 0.150), and a lower frequency of HLA-B*44 (10% vs. 16%, *P* = 0.008, *Pc* = 0.230) in pMBL/CLL than in healthy controls. However, only the differences with HLA-A*11 remained significant after Bonferroni correction (S2 Table). No statistically significant differences were found when non-pMBLs were compared with healthy donors or with pMBL/CLL patients.

The possible effect on 15-year progression to CLL of the described alleles was studied in the whole cohort of hiMBL in univariate analyses by the Kaplan–Meier, but this yielded no statistically significant differences between patients who carried or did not carry these alleles.

We then compared homozygosity among the three groups. We found no differences between overall homozygosity in non-pMBL and pMBL/CLL patients (17% vs. 15%, *P* = ns). Interestingly, we found higher overall homozygosity at all five HLA loci when non-pMBL (17% vs.11%, *P* = 0.020) and pMBL/CLL (15% vs.11%, *P* = 0.070) cases were compared with healthy donors. Checking for homozygosity at individual HLA loci revealed no differences between the three groups.

### Effect of HLA polymorphisms and clinical-biological variables on outcome of hiMBL and CLL

HLA allele frequencies were compared with respect to treatment requirement in accordance with IWCLL guidelines in the whole series including hiMBL and CLL. Higher frequencies of the HLA-A*24 (12% vs. 4%, *P* = 0.006, *Pc* = 0.110), HLA-DRB1*04 (19% vs. 11%, *P* = 0.038, *Pc* = 0.500), and HLA-C*03 (11% vs. 5%, *P* = 0.050, *Pc* = 0.730) alleles were found in treated patients than in untreated patients, although the differences were not maintained in any of the cases after Bonferroni correction (*Pc*>0.05; S3 Table).

When univariate Kaplan–Meier analyses were performed, the following variables were found to be associated with 15-year treatment requirement: 1) unmutated IGHV status (84% vs. 22%, *P*<0.0001), 2) intermediate/high-risk cytogenetics (85% vs. 38%, *P*<0.0001), 3) high β2-microglobulin level (67% vs. 38%, *P*<0.0001), 4) Binet A CLL diagnosis rather than hiMBL (56% vs. 27%, *P*<0.0001), 5) age ≤60 years (57% vs. 32%, *P*<0.01), and 6) HLA-A*24 (61% vs. 38%, *P* = 0.017) (see Table 2). In the multivariate analysis, unmutated IGHV status (HR: 5.3, 95% CI: 2.8–10.3), high β2-microglobulin level (HR: 5.1, 95% CI: 2.4–10.7), and age ≤60 years (HR: 2.0, 95% CI: 1.0–4.0) were significantly associated with treatment requirement. HLA-A*24 was significantly associated with the unmutated IGHV status (21% vs. 9%, *P* = 0.011) and intermediate/high-risk cytogenetics (30% vs. 14%, *P* = 0.033), which could explain the worse outcome of patients bearing this specificity.

**Table 2:** Univariate and multivariate analysis of factors influencing outcome in hiMBL and Binet A CLL individuals.

	Treatment requirement (n = 263)					Overall survival (n = 263)			
Variable	n	% 15-years	U	M	OR [95% CI]	% 15-years	U	M	OR [95% CI]
IGHV status									
<98% (mutated) *(Reference)*	179	22	1.4x10^-18^	5.4x10^-7^	5.3 [2.8–10.3]	84	5.0x10^-14^	1.1x10^-6^	9.9 [3.9–24.9]
≥98% (unmutated)	74	84				21			
β2-microglobulin									
≤3.5 mg/L *(Reference)*	212	38	1.8x10^-6^	1.6x10^-5^	5.1 [2.4–10.7]	63	0.0006	0.13	-
>3.5 mg/L	29	67				48			
Age									
≤60 years	72	57	0.0008	0.036	2.0 [1.0–4.0]	73	0.097	0.009	0.2 [0.07–0.7]
>60 years *(Reference)*	190	32				56			
CLL phenotype lymphocyte count									
hiMBL; ≤5.0 x10^9^/L CLL B cells (*Reference*)	155	27	3.6x10^-7^	0.063	1.9 [0.97–3.7]	67	0.034	0.22	-
CLL; >5.0 x10^9^/L CLL B cells	107	56				57			
HLA-A*24	32	61	0.017	0.8	-	46	0.11	0.84	-
Other HLA alleles *(Reference)*	224	38				65			

No significant differences in OS were found for any of the HLA alleles. Clinical variables influencing short OS in the multivariate analysis were unmutated IGHV status (HR: 9.9, 95% CI: 3.9–24.9), and age >60 years (HR: 4.6, 95% CI: 1.5–14.5) (Table 2).

### HLA polymorphisms and IGHV status

Since these results suggested that IGHV status significantly affects the outcome of hiMBL, we examined whether HLA influences IGHV status, and thereby hiMBL outcome.

We analyzed the effect of HLA with respect to IGHV status in the whole series including hiMBL and CLL (S4 Table). Biological and clinical characteristics of IGHV mutated and unmutated patients are compared in S5 Table.

Compared with their mutated counterparts, unmutated patients had higher allele frequencies of HLA-A*24 (11% vs. 4%, *P* = 0.016, *Pc* = 0.280), HLA-B*14 (12% vs. 3%, *P* = 0.001, *Pc* = 0.024), and HLA-C*08 (12.5% vs. 4%, *P* = 0.001, *Pc* = 0.014), and lower frequencies of HLA-C*04 (8% vs. 16%, *P* = 0.030, *Pc* = 0.400), and HLA-DQB1*03 (24% vs. 35%, *P* = 0.030, *Pc* = 0.160).

**Fig 1:**
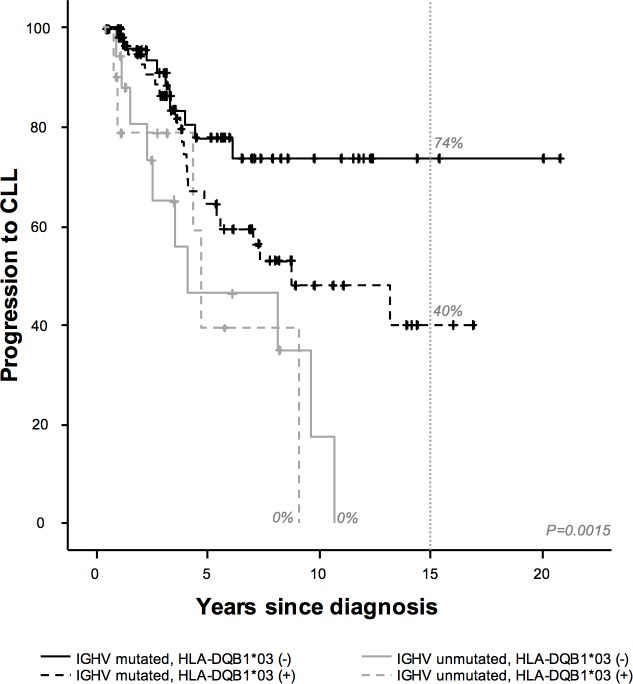
Kaplan–Meier analysis of 15-year hiMBL progression to CLL by HLA-DQB1*03 and IGHV status. IGHV mutated and unmutated status are depicted by black and gray lines, respectively. The presence and absence of HLA-DQB1*03 are shown by dashed and continuous lines, respectively. The vertical dashed line indicates 15-year follow-up.

Within the group of unmutated IGHVs (n = 74), no HLA alleles were associated with hiMBL progression to CLL, 15-year TFS or OS (data not shown). High β2-microglobulin level (100% vs. 76%, *P*<0.0001, HR: 6.8, 95% CI: 2.6–17.4), and age ≤60 years (100% vs. 69%, *P* = 0.009; HR: 2.7, 95% CI: 1.2–5.9) were significantly associated with 15-year treatment requirement, while only a high β2-microglobulin level (20% vs. 14%, *P*<0.001; HR: 4.2, 95% CI: 1.6–11.0) was associated with OS in this group. However, when we analyzed the mutated IGHV patients group, we found that those hiMBL (n = 121) with HLA-DQB1*03 (60% vs. 26%, *P* = 0.062, Fig 1), and those with >2.5x10^9^/L clonal B-cell count at diagnosis (88% vs. 35%, *P* = 0.001) had a higher risk of 15-year progression to CLL than those with other alleles. In the multivariate analysis, only the presence of a >2.5x10^9^/L clonal B-cell count (HR: 5.3, 95% CI: 1.6–17.6) was significantly associated with 15-year progression to CLL. Interestingly, those hiMBLs with HLA-DQB1*03 were significantly associated with the presence of >2.5x10^9^/L clonal B-cells (77% vs. 59%, *P* = 0.030). A difference in hiMBL progression to asymptomatic CLL with respect to HLA-DQB1*03 specificity was detected after five years.

**Fig 2:**
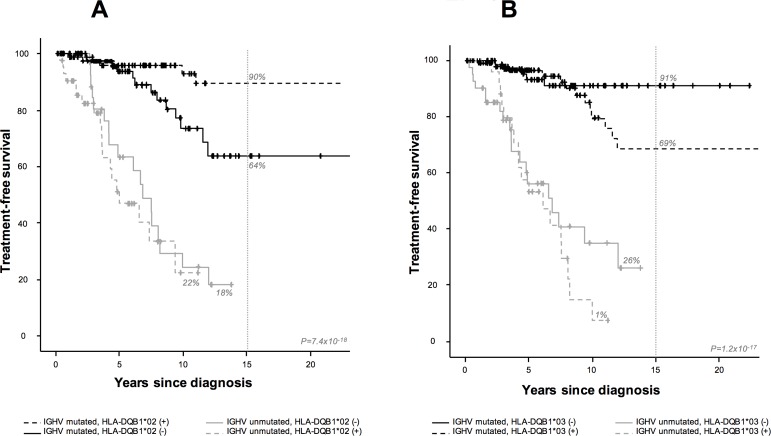
Kaplan–Meier analysis of 15-year treatment-free survival (TFS) by HLA alleles and IGHV status. (A) Kaplan–Meier analysis of 15-year treatment-free survival (TFS) by HLA-DQB1*02 and IGHV status (mutated vs. unmutated). (B) Kaplan–Meier analysis of 15-year treatment-free survival (TFS) by HLA-DQB1*03 and IGHV status (mutated vs. unmutated). IGHV mutated and unmutated status are depicted by black and gray lines, respectively. The presence and absence of the HLA-risk allele are shown by dashed and continuous lines, respectively. The vertical dashed line indicates 15-year follow-up.

Patients with mutated IGHV (n = 180) bearing the HLA-DQB1*02 allele had a lower 15-year treatment requirement (10% vs. 36%, *P* = 0.012, Fig 2A), whereas those with HLA-DQB1*03 allele had a higher 15-year treatment requirement (31% vs. 9%, P = 0.140, Fig 2B), although no statistical differences were found in the latter case. In the multivariate analysis, none of the factors was significantly associated with a high 15-year treatment requirement, although differences in the β2-microglobulin level (43% vs. 25%, *P* = 0.063, HR: 3.3, 95% CI: 0.94–11.5) were almost significant. OS was not evaluated due to the small number of events in this group (n = 9). Differences in treatment requirement with respect to HLA-DQB1*02 and HLA-DQB1*03 specificities were detected after a period of five and ten years, respectively.

## Discussion

hiMBL is known to be a precursor state of CLL. In fact, the distinction between hiMBL and CLL is based on an arbitrary threshold of 5x10^9^/L clonal B-cells [6, 9]. Thus, the identification of factors determining hiMBL progression to CLL and treatment requirement is a major challenge. Little information is available about clinical or genetic factors that determine hiMBL progression to CLL [4, 11, 12]. In recent years, several genome-wide association studies (GWAS) have described the 6p21.3 region as being a susceptibility risk region for both familial and sporadic CLL [14–17]. The HLA system, located in this region, plays an essential role in immunological surveillance [35].

Previous studies on CLL have shown significant, but inconsistent associations of certain HLA alleles such as DRB1*04:01, DQB1*03:02, and C*16 with the incidence of CLL [20, 26, 36]. The reason for such inconsistencies may reside in the small number of cases analyzed. More recent studies including larger series of patients have identified HLA-A*02:01, HLA-DRB4*01:01, as well as the haplotypes DRB4*01:01-DRB1*07:01-DQB1*03:03 and HLA-A*01-B*08-C*07 as being factors related to CLL predisposition [24, 26]. However, these were retrospective series based on patients previously identified as possible candidates for allogeneic stem-cell transplantation, which could have resulted in a bias arising from the inclusion of more severe diseases and younger patients.

Regarding the role of HLA in CLL outcome, the HLA-DRB1*01 allele has been linked to shorter treatment-free survival and OS, independently of the Rai clinical stage [25]. A major limitation of all the previous studies is that the analyses failed to consider the role of IGHV status, which clearly determines the outcome of CLL patients [1, 32] Given this and previous results, it is difficult to draw definite conclusions about the role of HLA in CLL, and there are no data available regarding their effect in hiMBL.

Here, we analyzed for the first time the influence of the HLA-A, -B, -C, -DRB1 and -DQB1 polymorphisms on the progression of hiMBL to CLL in a large series of 156 hiMBL consecutive individuals. In our series, no HLA specificities were significantly associated with hiMBL progression or OS. By contrast, clonal B-cell counts (>2.5x10^9^/L) and unmutated IGHV status were associated with shorter 15-year progression to asymptomatic CLL, as it has already been reported in prior studies [9, 12]. In addition, unmutated IGHV status, high β2-microglobulin level, and age ≤60 years were linked to shorter time to treatment requirement [9, 11, 12, 37].

Since these results suggest that IGHV status influences the outcome of hiMBL, we decided to analyze whether the HLA influences IGHV status, and thereby hiMBL outcome. No HLA specificities were correlated with hiMBL progression to asymptomatic CLL, treatment requirement or OS in unmutated IGHV patients, implying that this group of patients has a worse prognosis irrespective of the HLA system. Since the unmutated IGHV B-cell of CLL is considered to originate from an antigen-inexperienced naïve B cell, it is reasonable to suppose that the HLA system does not play a significant role in their behavior. On the other hand, the mutated IGHV CLL B-cells arise from a post-germinal center memory B cell that has required the HLA system to contact the antigen-presenting cell and to trigger the SHM. Considering these aspects, it seems possible that the mutated IGHV CLL B cells could have evolved from antigen-specific immune responses against prior infections or autoreactive processes [38–40].

One of the most relevant results in our study shows that, within the IGHV-mutated group, the presence of HLA-DQB1*03 is associated with a high risk of progression to CLL, while HLA-DQB1*02 is associated with longer treatment-free survival. The different abilities of particular alleles to efficiently present tumor antigens or the deregulation of allelic expression could lead to tumor cells escaping T cell and Natural Killer (NK) cell recognition [18, 41–46]. The HLA-DQB1*03 specificity was associated with a higher clonal B cell lymphocyte count at diagnosis, which may reflect a defective tumor antigen presentation and, therefore, progressive lymphocytosis. Alternatively, since mutated IGHV B cells have interacted with the antigen, those patients carrying the HLA-DQB1*03 specificity could produce a chronic activation of these B cells and, thereby, a greater susceptibility to acquiring genetic alterations, leading to the progression to CLL [47–49]. Furthermore, the detection after five years of differences in the mutated IGHV group with respect to the progression of hiMBL to asymptomatic CLL and treatment requirement suggests a chronic process. These findings also highlight the need for a very long follow-up to analyze factors that may influence hiMBL progression and treatment.

Conversely, the HLA-DQB1*02 allele is related to a lower treatment requirement, which could represent a protective allele in hiMBL/CLL, perhaps indicating that patients carrying this specificity are more able to control the CLL tumor clone. Previous studies have found a protective role of the A*01:01-C*07:01-B*08:01-DRB1*03:01-DQB1*02:01 haplotype in CLL patients [24]. However, we did not find any difference in the estimated frequency of this haplotype between treated and untreated patients (0.026 vs. 0.022, *P* = 0.700), independent of IGHV status (data not shown).

In summary, none of the previously reported associations of HLA polymorphisms with CLL was confirmed in our study. However, within the antigen-experienced mutated IGHV cohort, which represents the highest proportion of hiMBL, our results suggest a role for HLA in hiMBL prognosis. Thus, HLA-DQB1*03 could be a new marker of progression to CLL within mutated IGHV hiMBL that defines two risk groups, DQB1*03-positive as “medium-risk” and DQB1*03-negative as “low-risk”. Conversely, the HLA-DQB1*02 could be related to lower treatment requirement, and are consistent with the growing evidence of the influence of 6p21 on CLL predisposition. Larger non-biased series with long follow-up are required to enable definitive conclusions to be reached about the association of HLA with hiMBL/CLL development and outcome.

## Supporting information

S1 Table Univariate and multivariate analysis of factors influencing hiMBL progression to asymptomatic CLL. (DOC)

S2 Table HLA allelic frequencies comparing non-pMBL, pMBL/CLL, and healthy controls. (XLSX)

S3 Table HLA allelic frequencies comparing treated vs. untreated hiMBL and Binet A CLL patients. (XLSX)

S4 Table HLA allelic frequencies comparing mutated vs. unmutated hiMBL and Binet A CLL patients. (XLSX)

S5 Table Biological and clinical characteristics of mutated (n = 180) and unmutated (n = 74) cases at diagnosis. (DOC)
