# Supplementary material for: HLA specificities are associated with prognosis in IGHV-mutated CLL-like high-count monoclonal B cell lymphocytosis
Source: PLoS One. 2017 Mar 1;12(3):e0172978. doi: 10.1371/journal.pone.0172978 (PMC5332061; doi:10.1371/journal.pone.0172978)
Supplement: S1 Table — (DOC) [file pone.0172978.s001.doc]

Supplementary Table 1. Univariate and multivariate analysis of factors influencing hiMBL progression to asymptomatic CLL

|  | 15-year hiMBL progression to CLL (n=156) | | | | |
| --- | --- | --- | --- | --- | --- |
| **Variable** | **n** | **% 15-years** | **U** | **M** | **OR [95% CI]** |
| CLL phenotype lymphocyte count  ≤2.5 x10^9^/L CLL B cells  >2.5 x10^9^/L CLL B cells | 44  99 | 17  74 | 0.0003 | 0.006 | 3.7 [1.5-9.5] |
| IGHV status  <98% (mutated) *(Reference)*  ≥98% (unmutated) | 112  28 | 46  100 | 0.0004 | 0.005 | 2.6 [1.3-5.1] |
| HLA-A*11  Other HLA alleles *(Reference)* | 32  113 | 35  61 | 0.485 | - | - |
| HLA-DQB1*03  Other HLA alleles *(Reference)* | 73  74 | 66  43 | 0.117 | - | - |
| β2-microglobulin  ≤3.5 mg/L *(Reference)*  >3.5 mg/L | 130  17 | 55  70 | 0.531 | - | - |

Abbreviations: CI – Confidence interval, M – Multivariate analysis, NS – Not significant, OR – Odds ratio, U – Univariate analysis. Numbers in italics and underlined indicate *P*>0.05 and *P*<0.1, respectively.
