# Supplementary material for: HLA specificities are associated with prognosis in IGHV-mutated CLL-like high-count monoclonal B cell lymphocytosis
Source: PLoS One. 2017 Mar 1;12(3):e0172978. doi: 10.1371/journal.pone.0172978 (PMC5332061; doi:10.1371/journal.pone.0172978)
Supplement: S5 Table — (DOC) [file pone.0172978.s005.doc]

| **Supplementary Table 5**. Biological and clinical characteristics of mutated (n=180) and unmutated (n=74) cases at diagnosis | | | |
| --- | --- | --- | --- |
| **Variable** | **M n (%)** | **UM n (%)** | ***P*** |
| Median follow-up, months (range) | 97 (1-307) | 84 (6-239) | 0.123 |
| **Clinical variables at diagnosis** |  |  |  |
| Age [years, median (range)] | 66 (29-85) | 66 (30-89) | 0.934 |
| ≤60 | 47 (26) | 23 (31) | 0.256 |
| Sex |  |  |  |
| Male | 100 (56) | 48 (65) | 0.110 |
| WBC (mean ± SD; x10^9^/L) | 15.3±9.6 | 18.2±10.9 | 0.036 |
| Absolute lymphocyte count (mean ± SD; x10^9^/L) | 10.4±9.6 | 12.2±8.4 | 0.170 |
| CLL-type cells (mean ± SD; x10^9^/L) | 6.1±8.1 | 8.6±9.1 | 0.035 |
| β2-microglobulin >3.5 mg/L | 17 (10) | 11 (18) | 0.097 |
| Treated | 19 (11) | 39 (53) | 4.0x10^-12^ |
| **Biological variables at diagnosis** |  |  |  |
| FISH | *(n=156)* | *(n=63)* |  |
| Normal | 66 (42) | 25 (40) | 0.420 |
| del(11)(q22.3) | 0 (0) | 7 (11) | 0.0001 |
| +12 | 9 (6) | 16 (25) | 0.0001 |
| del(13)(q14) | 83 (53) | 20 (32) | 0.003 |
| del(17)(p13) | 3 (2) | 1 (2) | 0.672 |
